# A nomogram model for prognosis of acute ischemic stroke treated with recombinant tissue-type plasminogen activator

**DOI:** 10.3389/fneur.2025.1500534

**Published:** 2025-05-30

**Authors:** Song Bai, Yan Liu, Chenggang Zhang, Yuan Ren

**Affiliations:** ^1^Department of Neurosurgery, The First People’s Hospital of Chuzhou, Chuzhou, Anhui, China; ^2^Department of Neurology, The First People’s Hospital of Chuzhou, Chuzhou, Anhui, China

**Keywords:** acute ischemic stroke, thrombolysis, neutrophil inflammatory index, prognosis, nomogram model

## Abstract

**Objective:**

The primary objective of this study was to systematically assess the prognostic utility of neutrophil-derived inflammatory indices—specifically the neutrophil-to-lymphocyte ratio (NLR) and systemic inflammation response index (SIRI)—and subsequently develop a predictive model for thrombolysis outcomes through the integration of these hematological biomarkers with clinical parameters, including acute-phase neurological severity (as quantified by baseline National Institutes of Health Stroke Scale [NIHSS] scores) and therapeutic timeliness (door-to-needle time, DNT) in acute ischemic stroke patients receiving recombinant tissue plasminogen activator (rt-PA) therapy.

**Methods:**

This retrospective cohort study analyzed consecutive patients with AIS who received intravenous thrombolysis with rt-PA at the First People’s Hospital of Chuzhou in Anhui Province, China, between January 2021 and July 2023. Peripheral blood samples were collected within 24 h of admission to evaluate the NLR and SIRI. Univariate and multivariate logistic regression models were employed to assess the associations of NLR, SIRI, NIHSS score at admission, and DNT with short-term prognosis after intravenous thrombolysis for acute cerebral infarction. Receiver operating characteristic (ROC) curve analysis was conducted to assess the predictive capacity of the NLR, SIRI, NIHSS scores, DNT for clinical outcomes in AIS patients.

**Results:**

A total of 232 AIS patients treated with rt-PA IVT completed the 3-month follow-up. Multivariate logistic regression confirmed NIHSS score, DNT time, NLR, and SIRI as independently associated with worse prognosis (all *p* < 0.01). ROC analysis confirmed individual AUCs of 0.779 for NIHSS, 0.737 for NLR, 0.701 for SIRI, and 0.732 for DNT. The nomogram integrating NIHSS score, SIRI, NLR, and DNT was established, yielding an AUC of 0.885, and showing robust predictive accuracy with an internally validated AUC of 0.887 and externally validated AUC of 0.882.

**Conclusion:**

This study affirms that specific inflammatory markers like NLR and SIRI, alongside initial stroke severity and treatment timeliness, emerge as significant prognosis predictors in thrombolysis-treated stroke patients. By amalgamating these biomarkers, the proposed nomogram empowers clinicians to foresee and manage patient outcomes more effectively.

## Introduction

Acute Ischemic Stroke (AIS) represents a prominent contributor to long-term disability worldwide. Intravenous thrombolysis (IVT) employing alteplase stands as a well-established intervention significantly mitigating disability and mortality risks among AIS patients (1). Nevertheless, despite these advancements, some patients endure adverse functional outcomes stemming from intricate pathophysiological reactions triggered by reperfusion therapy (2, 3). Identifying prognosis-influencing factors, especially among those predisposed to poor outcomes post-treatment, remains paramount (4).

Stroke pathogenesis intricately intertwines with inflammatory cascades. Post-stroke immune responses and inflammation are recognized contributors to secondary brain damage post-reperfusion (4, 5). Recent investigations underscore the prognostic significance of neutrophils and associated inflammatory markers, such as the neutrophil-lymphocyte ratio (NLR), and the platelet–neutrophil ratio (PNR), in acute coronary syndrome (ACS) prognosis (6–9). Furthermore, emerging markers like the systemic immune inflammation index (SII) and systemic inflammation response index (SIRI), derived from routine blood counts, correlate with ACS outcomes (10). Notably, the documented association between SIRI and early functional deterioration in AIS underscores the significance of exploring the combined impact of SIRI and SII on AIS prognosis (11).

Neutrophil-mediated thromboinflammatory responses are increasingly recognized as a major contributor to blood–brain barrier disruption in patients with AIS following rt-PA reperfusion, leading to hemorrhagic transformation and secondary neurological deterioration (12). This study aims to integrate neutrophil-related inflammatory biomarkers with clinical parameters, such as DNT and NIHSS scores, to enhance and specify the clinical value of neutrophils. Furthermore, we have developed a novel model that allows clinicians to rapidly evaluate the short-term prognosis of patients with cerebral infarction following intravenous thrombolysis, optimize subsequent treatment strategies, and thus enhance the prognosis and clinical outcomes for individuals with AIS.

## Materials and methods

### Study design and population

**Figure 1:**
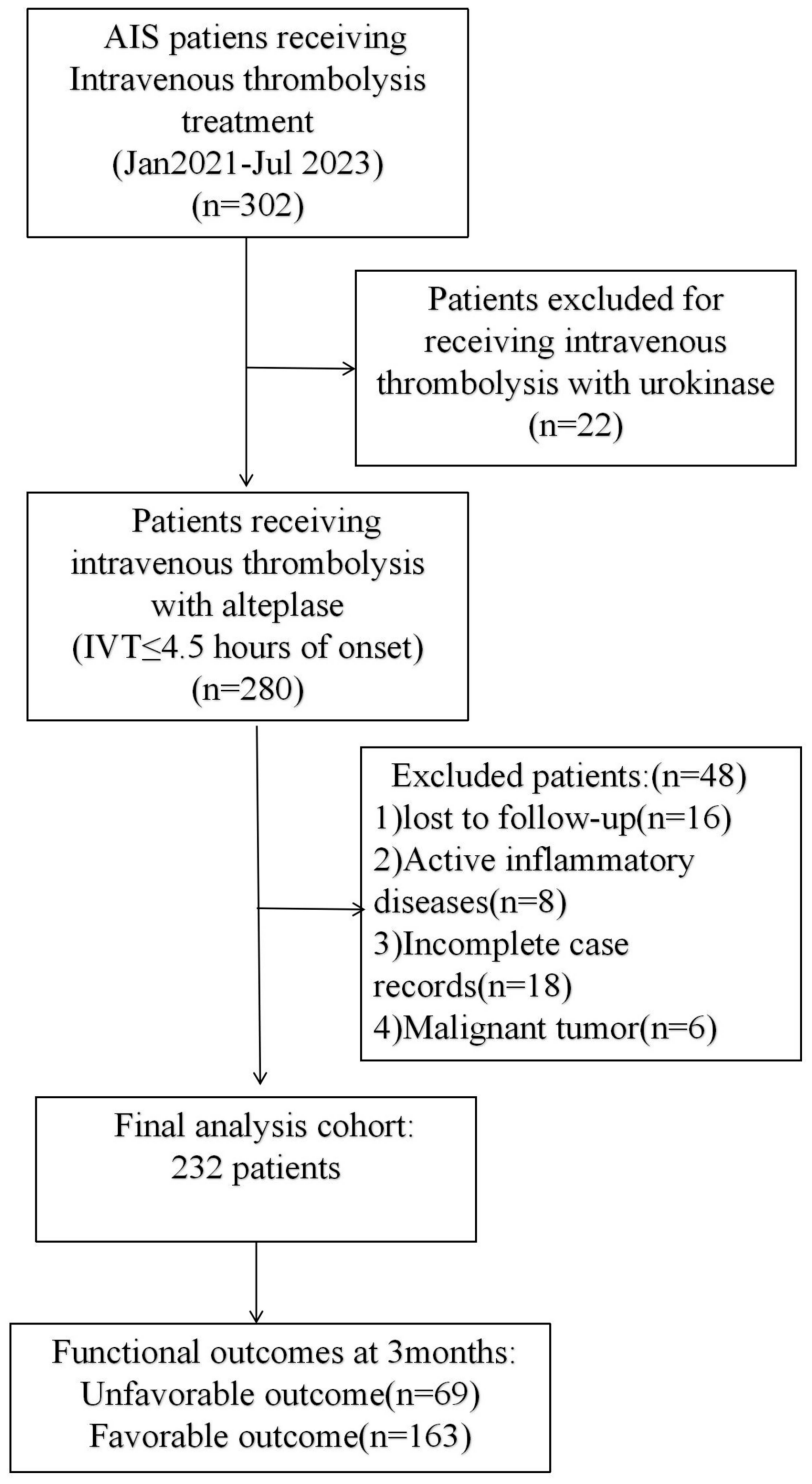
Study workflow chart.

This retrospective study encompassed AIS patients receiving intravenous thrombolysis (IVT) at The First People’s Hospital of Chuzhou between January 2021 and July 2023. The inclusion criteria were: (1) confirmed diagnosis of cerebral infarction via MRI or CT; (2) presentation within 4.5 h from symptom onset and treatment with rt-PA IVT; (3) availability of complete clinical data; and (4) informed consent obtained from the patient or their family. The exclusion criteria were: (1) presence of rheumatic or immune diseases; (2) malignant tumors; (3) recent acute myocardial infarction; (4) incomplete data; (5) severe liver or kidney damage; and (6) chronic inflammatory diseases. This study was approved by the Ethic Committee of the First People’s Hospital of Chuzhou. This article is a retrospective study. The patient selection process is depicted in Figure 1.

### Data collection and definition

Data were systematically collected from medical records, encompassing demographic information (age, gender), clinical history (atrial fibrillation, related diseases, smoking, alcohol consumption). And the patient has established cerebrovascular risk factors including hypertension (13) and diabetes mellitus (meeting international diagnostic criteria) (14). Hyperlipidemia diagnosis required meeting any one of these lipid threshold criteria: Total cholesterol (TC) ≥ 6.2 mmol/L; Low-density lipoprotein cholesterol (LDL-C) ≥ 4.1 mmol/L; Triglycerides (TG) ≥ 2.3 mmol/L; High-density lipoprotein cholesterol (HDL-C) < 1.0 mmol/L. The National Institutes of Health Stroke Scale (NIHSS) scores at admission, door-to-needle time (DNT), and 3-month post-thrombolysis modified Rankin Scale (mRS) score were independently assessed by two board-certified neurologists. Discrepancies in ratings (defined as ≥1-point difference in NIHSS/mRS or ≥5-min variance in DNT) were resolved through blinded consensus review by a third senior neurologist. Laboratory parameters included complete blood count (CBC) measurements obtained within 24 h of hospital admission. Four inflammatory biomarkers were calculated as follows: Neutrophil-to-Lymphocyte Ratio (NLR): neutrophil count/lymphocyte count; Systemic Inflammation Response Index (SIRI): (Neutrophil count × monocyte count)/lymphocyte count; Systemic Immune-Inflammation Index (SII): (Neutrophil count × platelet count)/lymphocyte count; Platelet-to-Neutrophil Ratio (PNR): Platelet count/neutrophil count.

The etiology of AIS was classified according to the TOAST criteria, which includes large artery atherosclerosis (LAA), small artery occlusion (SAO), and cardio-embolism (CE). All enrolled patients underwent head CT, MRI (including diffusion-weighted imaging, DWI), and CTA examinations.

Outcomes were assessed using the 3-month modified Rankin Scale (mRS), with patients categorized into two groups: a good outcome group (mRS score ≤ 2) and a poor outcome group (mRS score ≥ 3).

### Statistical analysis

Data analysis was performed using SPSS Statistics 26.0 and R software version 4.3.0. Continuous variables were expressed as either median (quartiles) or mean ± standard deviation, depending on their distribution, while categorical variables were presented as frequencies and percentages. Non-parametric tests were utilized for skewed data, and the chi-square or Fisher’s exact tests were applied to categorical variables as appropriate. Univariate logistic regression was conducted on all pertinent variables. Variables selected by the LASSO regression model were used in a multivariate logistic regression to develop a predictive model. The predictive accuracy of the inflammatory markers for adverse outcomes was assessed using Receiver Operating Characteristic (ROC) curve analysis.

## Results

### Baseline demographic characteristics

A total of 300 patients diagnosed with AIS and treated with rt-PA IVT within 4.5 h of onset were initially enrolled. After excluding 68 patients due to incomplete data, 232 participants completed a 3-month follow-up and were included in the study. Based on the modified Rankin Scale (mRS) scoring criteria, patients were categorized into a good prognosis group (mRS 0–2 points, *n* = 163) and a poor prognosis group (mRS 3–6 points, *n* = 69). The median age of the patients was 70 years, with a gender distribution of 62.07% male (144 cases) and 37.93% female (88 cases). There was no significant difference in age between the groups (*p* > 0.05), but a significant gender difference was observed, with males more likely to have a good prognosis (*p* < 0.05). A history of alcohol consumption was more common in the good prognosis group (*p* < 0.05). The median values of NIHSS score, DNT time, NLR, SIRI, SII, and neutrophil count were all significantly higher in the poor prognosis group (*p* < 0.001), and PNR also showed a significant difference between the two groups (*p* < 0.05). The proportions of hypertension and diabetes showed no significant differences (*p* > 0.05). Stroke subtype appeared to influence the prognosis (*p* < 0.05) (Table 1).

**Table 1:** General clinical characteristics of AIS patients receiving rt-PA treatment in the good and poor function outcomes groups.

Variable	Total (*n* = 232)	Functional outcomes		*P*
		mRS 0–2 (*n* = 163)	mRS 3–6 (*n* = 69)	
Demographic data				
Age (years)	70.00 (57.00–79.00)	70.00 (55.00–79.00)	71.00 (59.00–79.00)	0.096
Sex (male, *n*, %)	144 (62.07)	111 (68.10)	33 (47.83)	0.004
Stroke risk factors (*n*, %)				
Smoking	66 (28.45)	50 (30.67)	16 (23.19)	0.248
Alcohol	35 (15.09)	30 (18.40)	5 (7.25)	0.030
Hypertension (*n*, %)	183 (78.88)	127 (77.91)	56 (81.16)	0.580
Diabetes (*n*, %)	67 (28.88)	46 (28.22)	21 (30.43)	0.734
Hyperlipidemia (*n*, %)	35 (15.09)	26 (15.95)	9 (13.04)	0.572
Atrial fibrillation (*n*, %)	37 (15.95)	21 (12.88)	16 (23.19)	0.050
CAD (*n*, %)	29 (12.5)	21 (12.88)	8 (11.59)	0.786
Prior stroke (*n*, %)	40 (17.24)	32 (19.63)	8 (11.59)	0.138
Clinical features				
NIHSS on admission	4.00 (3.00–9.00)	4.00 (2.00–6.00)	10.00 (5.00–16.00)	<0.001
DNT	58.00 (42.00–90.00)	50.00 (40.00–75.00)	90.00 (56.00–120.00)	<0.001
Serum biomarkers				
Neutrophil	4.41 (3.22–6.23)	3.92 (3.02–5.66)	5.33 (3.84–9.10)	<0.001
NLR	3.11 (1.71–4.92)	2.65 (1.50–4.18)	4.91 (2.87–7.75)	<0.001
PNR	39.28 (26.04–56.83)	44.25 (30.03–62.36)	31.40 (21.01–44.80)	<0.001
SIRI	1.17 (0.59–2.39)	0.98 (0.51–1.82)	2.37 (1.08–3.38)	<0.001
SII	600.96 (292.91–1103.52)	470.62 (269.64–840.18)	1182.50 (473.53–2077.13)	<0.001
TOAST subtype (*n*, %)				0.020
Large artery atherosclerosis (*n*, %)	174 (75)	119 (73.01)	55 (79.71)	
Small vessel occlusion (*n*, %)	31 (13.36)	28 (17.18)	3 (4.35)	
Cardioembolism (*n*, %)	27 (11.64)	16 (9.82)	11 (15.94)	

mRS, modified ranking scale; *n*; CAD, coronary artery disease; DNT, door to needle time; NLR, neutrophil to lymphocyte ratio; PNR, Platelet neutrophil ratio; SIRI, systemic inflammation response index; SII, systemic inflammatory reaction index.

### Logistic regression analysis of AIS patients after 3 months of IVT treatment

**Figure 2:**
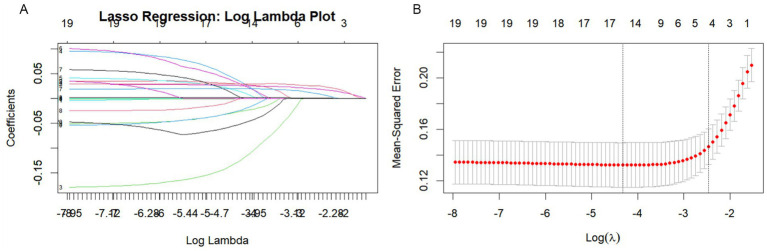
Lasso regression analysis based on Selection of predictors. **(A)** Lasso regression incorporated all the provided variables (NIHSS, SIRI, NLR, DNT) as features for the predictive model. **(B)** Cross-validation curve for LASSO regression analysis.

Univariate binary logistic regression analysis (Supplementary Table S1) found that gender, alcohol consumption, NIHSS score at admission, DNT time, PNR, NLR, SII, and SIRI as significantly associated with adverse outcomes three months post-stroke (all *p* < 0.05). LASSO regression analysis was conducted to refine the selection of predictors, identifying four key variables: NIHSS score at admission, DNT, SIRI, and NLR (Figure 2). Multivariate logistic regression analysis revealed that NIHSS score at admission (OR = 1.21, 95% CI: 1.12–1.30, *p* < 0.001), DNT time (OR = 1.01, 95% CI: 1.01–1.02, *p* = 0.009), NLR (OR = 1.28, 95% CI: 1.13–1.46, *p* < 0.001), and SIRI (OR = 1.31, 95% CI: 1.07–1.59, *p* = 0.008) were associated with a worse prognosis (Supplementary Table S2).

### Establishment of nomogram model

**Figure 3:**
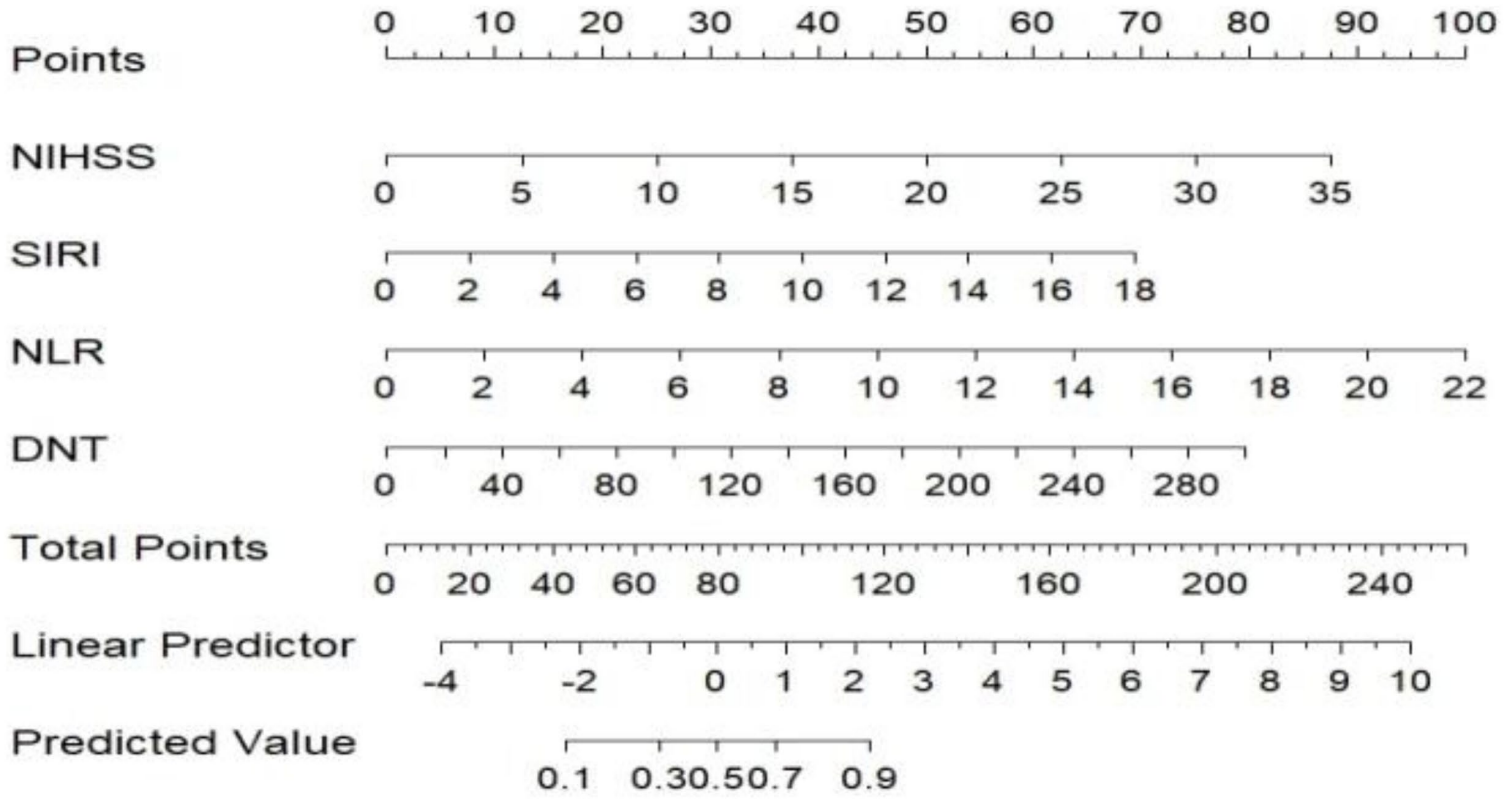
Predictive model for adverse outcomes after 3 months of rt-PA intravenous thrombolysis (Nomogram). NIHSS, National Institute of Health Stroke Scale; SIRI, systemic inflammation response index; NLR, neutrophil to lymphocyte ratio; DNT, door to needle time.

**Figure 4:**
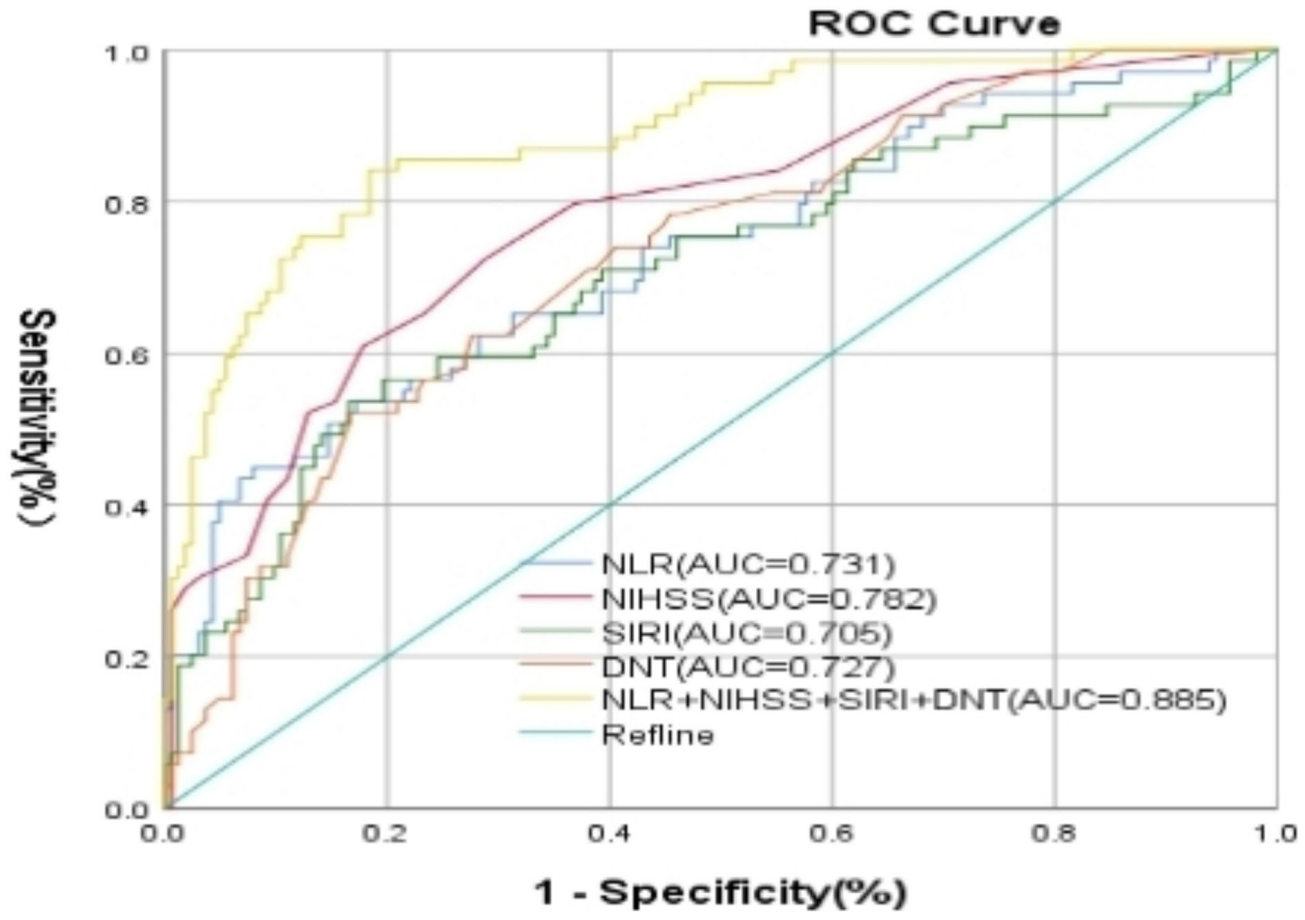
Characteristics of subject ROC curves for NIHSS score, DNT, NLR, and NLR + SIRI, and NIHSS + DNT + SIRI + NLR on prognosis of patients with AIS undergoing intravenous thrombolysis at admission. NIHSS, National Institute of Health Stroke Scale; SIRI, systemic inflammation response index; NLR, neutrophil to lymphocyte ratio; DNT, door to needle time.

**Figure 5:**
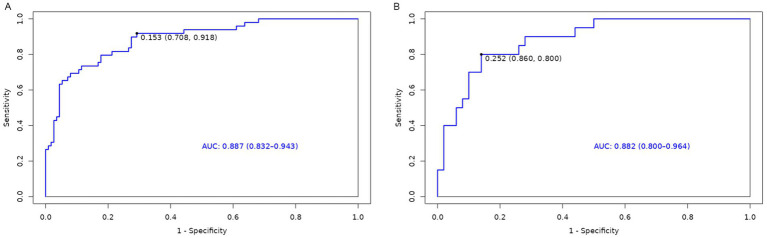
AUC of training and validation group. **(A)** Training Set. **(B)** Validation Set.

**Figure 6:**
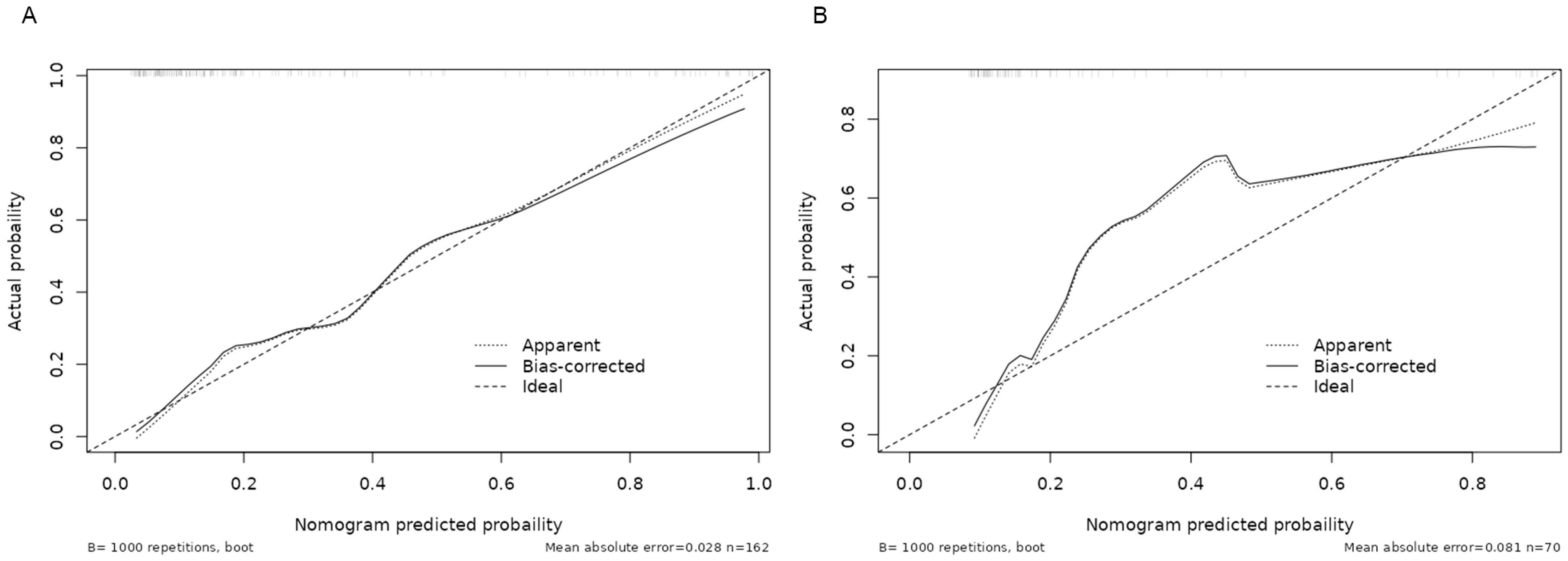
Calibration curve of the nomogram for the risk of adverse outcomes. **(A)** Training cohort. **(B)** Validation cohort.

**Figure 7:**
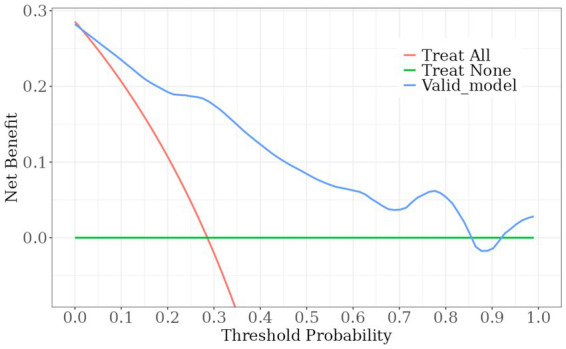
DCA curve. Decision curve assessment of the poor prognosis prediction model. The red line indicates the assumption that all patient outcomes were poor prognosis and interventions were implemented ahead of time; the green line indicates that no patient experienced a poor outcome and no interventions were implemented ahead of time; and the blue line indicates the constructed risk-nomogram for poor prognosis.

A nomogram model was developed using the key independent predictors identified (NIHSS score, SIRI, NLR and DNT) and represented in a nomogram to estimate the probability of an adverse outcome (Figure 3). ROC Curve Analysis assessed the predictive ability of NIHSS score at admission, DNT time, NLR, and SIRI for adverse outcomes. The AUC values were as follows: NIHSS score at admission 0.779, SIRI 0.701, NLR 0.737, and DNT 0.732. The combined index of NIHSS, DNT, NLR, and SIRI yielded an AUC of 0.885, indicating a strong predictive model (Figure 4). The model demonstrated robust predictive accuracy with an internally validated AUC value of 0.887 (95% CI: 0.832–0.934) and an externally validated AUC value of 0.882 (95% CI: 0.800–0.964), both exceeding the threshold AUC value of > 0.7 (Figure 5). Calibration curves for internal and external validation showed high concordance with the ideal curve, confirming the model’s reliability (Figure 6). The decision curve analysis indicated that when the probability threshold for unfavorable outcomes is between 0.04 and 0.86, patients with acute cerebral infarction may benefit from earlier interventions (Figure 7).

## Discussion

This study significantly enhances our understanding of predictive factors for short-term adverse outcomes in AIS patients treated with IVT. By integrating multiple predictors-specifically, the NIHSS score at admission, DNT, NLR, and SIRI-our research demonstrates the superior predictive power of combining clinical features and inflammatory markers over traditional single-marker models. The developed predictive nomogram, achieving an AUC value of 0.885, effectively identifies patients at high risk of poor outcomes, enabling clinicians to tailor interventions more precisely and potentially improve patient management and outcomes. This approach not only underscores the importance of rapid treatment and comprehensive assessment in AIS but also sets the stage for future research to optimize and personalize stroke treatment strategies.

Current research on predicting short-term adverse outcomes after IVT has primarily focused on individual inflammatory markers, with limited analysis of NLR, PNR, SII, SIRI, DNT, and NIHSS score in combination. The objective of this study was to evaluate the predictive value of NLR, SIRI combined with NIHSS score, and DNT time on the prognostic outcome of IVT. Our findings indicate that elevated SIRI, NLR, NIHSS score, and DNT time were significantly associated with poor functional prognosis in patients. Even after adjusting for potential confounders, high SIRI remained significantly correlated with poor outcomes. Utilizing these metrics, we developed a novel Nomogram (15) to predict short-term prognosis for IVT patients at risk of adverse outcomes.

Multiple investigations have demonstrated that three months following IVT, the NLR can predict the functional result (7, 9). High NLR values could predict negative results (7). Circulating neutrophils promote disruption of the blood–brain barrier and increase brain tissue damage after ischemia (16). Histological studies have shown that neutrophils are associated with plaque formation in carotid atherosclerosis (16). T-lymphocytes act as preventive immunomodulators in the brain and can be significant in acute stroke, but they also increase the risk of ischemia–reperfusion injury (17). In addition to their complicated, interconnected roles in the removal of dead tissues, neutrophils, monocytes, microglia, T and B lymphocytes, and other immune cells can create persistent inflammation and inadvertently damage healthy brain cells, both of which impact the prognosis of stroke (18). The ratio of neutrophils to lymphocytes reflects the immune status, and even inflammatory cytokines secreted by neutrophils can trigger lymphocyte apoptosis (17). The present study corroborates the findings of previous research.

Some studies have found that systemic immune-inflammation index (SII), an indicator of systemic inflammation, is closely associated with the prognosis of malignant tumors (19, 20). Clinical studies have reported that SII can predict coronary artery disease (21, 22) and is correlated with short-term adverse prognosis of ischemic stroke (23), providing valuable clinical reference for assessing adverse prognosis. Another systemic inflammatory marker, SIRI, has been associated with prognosis after coronary vascular stenting (24) and recently linked to poor prognosis in ischemic stroke (25). In the present study, SIRI demonstrated better predictive ability than SII for poor prognosis after IVT. The likely reason for this is that SII includes platelets, unlike SIRI, which includes monocytes. Monocytes (and their derived macrophages) play an increasingly vital role in the development and propagation of atherosclerotic plaques (26). They not only participate in the initial formation of plaques but also influence plaque stability and susceptibility to rupture. A key feature of the inflammatory response following cerebral ischemia is the infiltration of monocytes (27). Platelets, conversely, are more active in the early stages of the disease, particularly in the initial formation of plaques and the stimulation of the inflammatory response (28). Monocytes primarily participate in tissue healing and the inflammatory response. Higher SIRI reflects intense pro-inflammatory reactivity generated by monocytes, neutrophils, and lymphocytes, correlated with elevated neutrophil/monocyte and/or lower lymphocyte numbers (29). Sensitivity and specificity analyses revealed that combining NLR, SIRI with DNT and NIHSS scores to create a new index improves sensitivity and specificity compared to NLR or SIRI alone. This biomarker can be easily calculated. Inflammation is a major contributing factor to thrombosis and ischemic brain tissue damage, and the combined application of inflammatory markers may more comprehensively assess patient prognosis. Thus, aiding physicians in early and relatively precise prognostic estimation for AIS patients.

While this study offers valuable insights into the predictive value of combined clinical and inflammatory markers in stroke prognosis, it has limitations. Being retrospective, it is susceptible to selection bias, and the findings rely on a relatively small sample size, potentially limiting generalizability. To address these limitations, we are currently expanding our data collection efforts prospectively. This includes increasing the sample size and extending beyond our hospital to improve the validity and clinical utility of our predictive model. Our future work aims to validate our findings in a larger, more diverse cohort, potentially strengthening the clinical applicability of our novel predictive nomogram.

In conclusion, the NIHSS score at admission, DNT time, and the neutrophil-based inflammatory indexes NLR and SIRI are effective predictors of poor prognosis following IVT in patients with AIS. Combining NLR and SIRI with the NIHSS score and DNT enhances the accuracy and reliability of predicting adverse outcomes. This integrated approach enables a more personalized and tailored treatment strategy compared to relying on a single index alone.

## Data Availability

The original contributions presented in the study are included in the article/Supplementary material, further inquiries can be directed to the corresponding author.
