# Supplementary material for: A nomogram model for prognosis of acute ischemic stroke treated with recombinant tissue-type plasminogen activator
Source: Front Neurol. 2025 May 30;16:1500534. doi: 10.3389/fneur.2025.1500534 (PMC12164161; doi:10.3389/fneur.2025.1500534)
Supplement: Supplementary file 1 [file Table_1.DOCX]

**Table S1. Univariate logistic regression analysis for 3-month poor outcome.**

| **Variables** | **OR (95% CI)** | ***P*** |
| --- | --- | --- |
| Age (years) | 1.02 (1.00 - 1.04) | 0.077 |
| Sex (male, n%) | 0.43（0.24-0.76） | 0.004 |
| Smoking | 1.47（0.77-2.81） | 0.249 |
| Alcohol | 2.89(1.07-7.79) | 0.036 |
| Hypertension | 0.82（0.40-1.66） | 0.580 |
| Diabetes | 0.90（0.49-1.66） | 0.734 |
| AF | 0.50（0.24-1.10） | 0.053 |
| CAD | 1.13（0.47-2.69） | 0.786 |
| Prior stroke | 1.86（0.81-4.28） | 0.143 |
| NIHSS on admission | 1.21 (1.13 - 1.29) | <0.001 |
| DNT | 1.02 (1.01 - 1.03) | <0.001 |
| Serum biomarkers |  |  |
| Neutrophils (10^9^/L) | 1.17 (1.09 - 1.25) | <0.001 |
| PNR | 0.98 (0.96 - 0.99) | 0.001 |
| NLR | 1.37 (1.22 - 1.54) | <0.001 |
| SII | 1.01 (1.01 - 1.01) | <0.001 |
| SIRI | 1.50 (1.25 - 1.81) | <0.001 |
| Stroke subtype, n (%) |  |  |
| LAA |  | 1.00 (Reference) |
| SAO | 0.230 (0.07 - 0.80) | 0.020 |
| CE | 1.49 (0.65 - 3.42) | 0.349 |

Abbreviations: AF, atrial fibrillation; CAD, coronary artery disease; NIHSS, national institute of health stroke scale; DNT, door to needle time; NLR,neutrophil to lymphocyte ratio; PNR, Platelet neutrophil ratio; SIRI, systemic inflammation response index; SII, systemic inflammatory reaction index; LAA, large artery atherosclerosis; SAO, small artery occlusion; CE, cardio-embolism.

**Table S2. Multivariate logistic regression analysis for 3-month poor outcome.**

| **Variables** | **OR (95%CI)** | ***P*** |
| --- | --- | --- |
| NIHSS | 1.21 (1.12 - 1.30) | <.001 |
| DNT | 1.01 (1.01 - 1.02) | 0.009 |
| NLR | 1.28 (1.13 - 1.46) | <.001 |
| SIRI | 1.31 (1.07 - 1.59) | 0.008 |

Abbreviations: NIHSS, national institute of health stroke scale; DNT, door to needle time; NLR, neutrophil to lymphocyte ratio; SIRI, systemic inflammation response index.
